# Proteomic Profiling of *Ex Vivo* Expanded CD34-Positive Haematopoetic Cells Derived from Umbilical Cord Blood

**DOI:** 10.1155/2013/245695

**Published:** 2013-03-26

**Authors:** Heiner Falkenberg, Teja Falk Radke, Gesine Kögler, Kai Stühler

**Affiliations:** ^1^Molecular Proteomics Laboratory (MPL), Center for Biomedical Research (BMFZ), Heinrich Heine University, Universitätsstrasse 1, 40225 Düsseldorf, Germany; ^2^Institute for Transplantation Diagnostics and Cell Therapeutics, Heinrich Heine University Medical Center, 40225 Düsseldorf, Germany

## Abstract

*Ex vivo* expansion of haematopoetic cells by application of specific cytokines is one approach to overcome boundaries in cord blood transplantation due to limited numbers of haematopoetic stem cells. While many protocols describe an effective increase of total cell numbers and the amount of CD34-positive cells, it still remains unclear if and how the procedure actually affects the cells' properties. In the presented publications, CD34-positive cells were isolated from cord blood and expanded for up to 7 days in media supplemented with stem cell factor (SCF), thrombopoietin (THPO), interleukin 6 (IL-6), and fms-related tyrosine kinase 3 ligand (FLT3lg). At days 3 and 7, expanded cells were harvested and analyzed by flow cytometry and quantitative proteomics. 2970 proteins were identified, whereof proteomic analysis showed 440 proteins significantly changed in abundance during *ex vivo* expansion. Despite the fact that haematopoetic cells still expressed CD34 on the surface after 3 days, major changes in regard to the protein profile were observed, while further expansion showed less effect on the proteome level. Enrichment analysis of biological processes clearly showed a proteomic change toward a protein biosynthesis phenotype already within the first three days of expression.

## 1. Introduction

Although several groups in preclinical and clinical settings have attempted *ex vivo* expansion of the cord blood (CB) product in order to increase haematopoetic progenitor and granulocyte numbers and to reduce the duration of posttransplant neutropenia (summarized in [1]), the implementation of protocols applying for instance several cytokines has been complicated by the following facts.

1. CB transplants are frozen in the majority of banks in a single bag. Clinical trials were performed with only a fraction of CB unit expanded *ex vivo* with the larger remainder infused unmanipulated. Therefore, the expanded product usually could be infused only 10–14 days after transplantation. Alternative approaches focus on the expansion of one CB unit together with a second nonmanipulated unit.
2. Clinical grade growth factors are only available for a limited number of cytokines and are expensive.
3. Moreover, none of the clinical experiences could unequivocally document a clear benefit of infusion of such *ex vivo* cytokine expanded components.

Since cytokine-driven *ex vivo *expansion of CD34^+^ cells from CB is being discussed controversially, other ways to improve haematopoetic engraftment time and reconstitution after CB transplantation are being explored including double CB transplants (summarized in [2]) and cotransplantation of a single CB unit together with highly purified CD34^+^ mobilized peripheral blood stem cells from a haploidentical or related or completely unrelated donor as a very promising clinical approach [3].

Recently Csaszar et al. presented an interesting approach for rapid expansion of human HSC by automated control of inhibitory feedback mechanism [4], whereas the group of Delaney et al. is focusing on Notch-mediated expansion [5]. Although the CD34^+^ expanded cell populations were well characterized, no clear protein data are available to define the changes in the CD34^+^ population in the expanded product.

Mass spectrometry (MS)-based proteomics allows rapid assessment of changes in protein expression [6]. However, in spite of its potential, only few studies applied proteomics to obtain insights into biological processes within cord-blood-derived CD34^+^ cells [7–12]. With a broad spectrum of different techniques, analysis of cell lysate led to the identification of up to 370 proteins [10] in native CD34^+^ cord blood cells. Nevertheless, biological processes occurring during *ex vivo* expansion need further elucidation.

While these studies were limited to a detection of qualitative description of native CD34^+^ cells, emerging techniques are in the position to identify more than thousands of proteins within a cell and also to quantify these proteins in parallel. In contrast to labeling-based quantification techniques such as ICAT, iTRAQ, TMTs or SILAC, label-free quantification avoid any additional sample preparation step and allows direct analysis of clinical specimen [13–15]. Therefore, a label-free approach was chosen to profile expression changes of *ex vivo* expanded CD34^+^ haematopoetic stem/progenitor cells derived from umbilical cord blood.

This is the first report applying label-free proteomics to reveal proteomic changes during *ex vivo *expansion of CD34^+^ haematopoetic stem/progenitor cells derived from umbilical cord blood. The results clearly document significant changes towards a protein biosynthesis phenotype already 3 days after expansion not reflected by the immunophenotype. 

## 2. Material and Methods

### 2.1. Collection of Cord Blood

CB was collected with the informed consent of the mother according to established methods [16]. Briefly, after delivery of the baby, the cord was doubly clamped and transected, and the blood was collected in special collection bags containing citrate-phosphate dextrose. In the experiments performed, only CB units not suitable for banking due to exclusion criteria, such as low cell number or low volume, were used (ethics committee approval no. 2975).

### 2.2. Isolation of CD34^+^-Positive Haematopoetic Cells

CD34-positive cells were isolated using a two-step procedure of magnetic activated cell sorting (MACS) and fluorescence activated cell sorting (FACS).

Firstly, mononuclear cells were isolated using Ficoll-based density centrifugation (Biochrom; Berlin, Germany) as described in previous publications [17]. Remaining erythrocytes were removed by ammonium-chloride lysis (10 min.; 4°C), and cells were washed twice with phosphate buffered saline. Subsequent enrichment of CD34^+^ cells was performed by antibody labeling with paramagnetic beads against the CD34-epitope (Indirect CD34 Micro Bead Kit) and by application of LS-columns and MIDI-MACS magnets (all Miltenyi; Bergisch Gladbach, Germany) according the manufacturer's instructions. 

Secondly, in order to achieve 100% purity, the enriched cells (after one MACS-column) were stained with antibodies against CD34 (PE-conjugated) and CD45 (FITC-conjugated; both BD Biosciences; San Jose, USA) and sorted for the surface expression of CD34^high^/CD45^low^ using a MoFlo XDP cell sorter (Beckman Coulter; Fullerton, USA) at the Core Flow Cytometry Center at the Düsseldorf University Medical Centre.

### 2.3. Expansion of CD34^+^-Positive Haematopoetic Cells

The isolated CD34^+^ cells were seeded at a density of 2*10^5^/3 ml/well on 6-well plates and incubated at standard cell culture conditions (37°C, 5% CO_2_, humidified air) in expansion media consisting of DMEM (Lonza; Basel, Switzerland) with 30% fetal calve serum (Gibco; Karlsruhe, Germany), penicillin/streptomycin/glutamine (all Lonza), and cytokines (50 ng/mL SCF, 20 ng/mL FLT3 lg, 50 ng/mL IL-6, 10 ng/mL TPO; all R&D Systems; Minneapolis, USA). On days 3 and 7, respectively, total cells were harvested, enumerated by Neubauer-chamber, and, after washing with PBS two times, used for protein isolation. In parallel, an aliquot of app. 5*10^4^ cells was stained against CD34/CD45 for flow cytometric analysis (FACSCanto; BD Biosciences).

### 2.4. Cell Lysis and Sample Preparation

For protein extraction, cells were lysed and homogenised in lysis buffer (2 M thiourea, 7 M urea, 30 mM Tris-HCl, pH 8.0). After determination of protein concentration using Bradford assay 10 *µ*g of proteins were loaded on a SDS-PAGE. After complete entry into the gel, the SDS-PAGE was stopped after 5 min. Gels were silver stained according to [18] and one band per sample was cut out. Bands were destained and washed, and proteins were digested with trypsin (Promega, Mannheim, Germany).

### 2.5. Mass Spectrometric Analysis

Extracted peptides were analysed in a shuffled batch design with a HPLC (RSLCnano U3000, Thermo Fisher Scientific, Bremen, Germany) online coupled to a LTQ Orbitrap Elite (Thermo Fisher Scientific) mass spectrometer.

Samples were loaded on a trap column (Acclaim PepMap C18; 2 cm × 100 *µ*m × 5 *µ*m, 100 Å, Thermo Fisher Scientific) and washed with 0.1% TFA for 10 minutes. Trap column was switched online with separation column (Acclaim PepMap RSLC C18; 25 cm × 75 *µ*m × 2 *µ*m, 100 Å, Thermo Fisher Scientific) and peptides were separated for 120 minutes using a gradient of A: 0.1% FA and B: 0.1% FA, 84% ACN. The gradient started with 4% B and rose up to 40% B, followed by a washing step with 95% B for 5 minutes. Peptides were ionized via electrospray (1.2 keV). During chromatographic separation, the LTQ Orbitrap Elite was operated in a TOP20 data-dependent mode. MS-spectra were measured in the Orbitrap, mass range 350–1700 *m*/*z*, polysiloxane (445.120030 Da) as lock mass. From up to 20 most intense ions MS/MS spectra after collision induced dissociation in the iontrap were acquired. Dynamic exclusion of already measured ions was enabled.

### 2.6. Identification and Quantification of Proteins

For peptide and protein identification as well as for label-free quantification we used MaxQuant (Version 1.2.7.4) associated with Andromeda [19, 20]. MS/MS spectra were searched against the Uniprot/Swissprot-Database (human entries only, date 13/06/2012) with a mass tolerance of 10 ppm or 0.5 Da in MS and MS/MS mode, respectively. Oxidation of methionine as well as N-terminal acetylation as variable modifications were considered during database search. The false discovery rate (FDR) on peptide and protein level was below 1%. Known contaminants and reverse entries were removed from the protein list.

A minimum of 2 unique peptides were required for identification as well as for quantification. All proteins were quantified only based on unique peptides. Furthermore, only proteins identified in all experiments were regarded for quantification.

Statistical analysis was performed using Perseus as part of MaxQuant. R-based two sample *t*-tests (FDR < 0.05, *S*0 = 0.8) between all conditions were used to declare a protein regulation as significant. log⁡_2_-expression values were plotted against the *P* value for graphical clarification of significant protein regulation (Figure 3). Fisher's exact test was used to extract enriched GO-Terms, KEGG-Pathways, and Uniprot-Keywords (*P* value <0.001, enrichment > 2.3).

A list with all identified proteins, peptides (included modified peptides), and label-free quantification values with detailed quantification parameter is presented in supplementary Table  2 (see supplementary materials available online at http://dx.doi.org/10.1155/2013/245695).

## 3. Results 

The application of cord blood cells for clinical trials and transplantation is often limited by low amount of CD34^+^ haematopoetic stem cells [1]. Therefore, *ex vivo* expansion is often used to increase the number of CD34^+^ cells. To monitor changes of CD34^+^ cells during *ex vivo* expansion we chose a proteomic approach allowing us to profile changes on protein level and give insight into altered biological processes. Here, we analysed CD34^+^ cells isolated under GMP-conditions and *ex vivo* expanded for up to seven days. Whole cell lysates of FACS-isolated CD34^+^ cells (day 0) and subsequently expanded cells were analysed using label-free MS analysis of day 3 and day 7, respectively. 

### 3.1. *Ex Vivo* Expansion of CD34^+^ Haematopoetic Stem/Progenitor Cells

**Figure 1:**
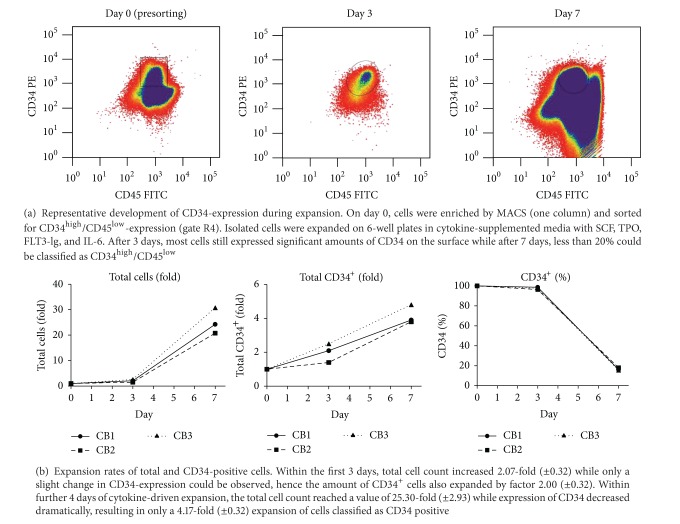
Umbilical cord blood cells were enriched by MACS and sorted for CD34^high^/CD45^low^-expression.

Following expansion in cytokine-supplemented media (SCF, TPO, FLT3-lg, IL-6) cell numbers of CD34^+^ haematopoetic stem/progenitor cells increased by factors 2.1 and 4.2 after 3 and 7 days, respectively (Figure 1(b)). It is interesting to note that after 3 days the CD34^+^ cells mainly dominate the cell population (amount of CD34^+^ cells: 97.5%), whereas the CD34^+^ cells reflected by a total increase of cell number by factor 25.3 were underrepresented after 7 days (amount of CD34^+^ cells: 16.6%). As preliminary experiments revealed, appliance of this expansion protocol also increased the amount of colony-forming units approximately by factor 2 on day 3 and by factor 6 on day 7, respectively (supplementary Figure 1).

### 3.2. Proteome Profiling of *Ex Vivo* Expansion

For proteome profiling we exploit the advantage of label-free proteome analysis without prior protein labelling allowing us to quantify and identify complex protein mixtures on the same analytical platform. The whole cell lysates of three CB donors isolated at day 0 (after withdrawal and FACS enrichment), day 3, and day 7 after *ex vivo* expansion were analysed. Altogether we identified 2970 unique proteins. Detailed Gene Ontology (GO) annotation showed that we identified proteins from almost all cellular compartments (nucleus, cytosol, Golgi apparatus, cytoskeleton, membrane, extracellular region, and more) involved in 65 different biological processes (based on GO slim terms, data not shown). For further analysis of proteome changes during *ex vivo* expansion we only considered 1343 proteins quantified in all three donors and time points.

**Figure 2:**
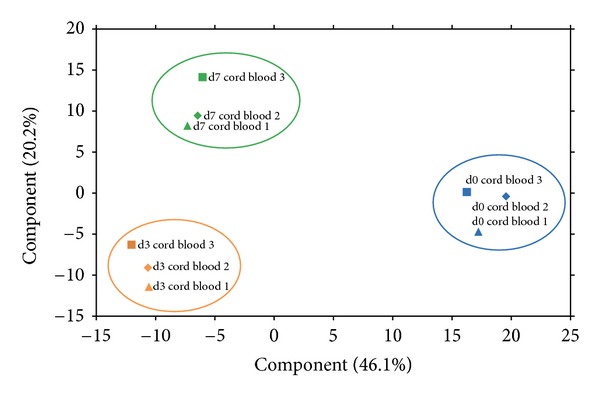
Principal component analysis (PCA) of CD34^+^ cells, showing that the three time points are distinct. Based on protein expression data, the largest variance was observed between day 0 and day 3; compared to this, the variation between day 3 and day 7 is smaller. Only little variances were observed between the three different cord blood donors.

Principal compound analysis (PCA) clearly demonstrates that the greatest variance of the datasets was obtained from the analysed time points and not from individual donors (Figure 2). By analysing the reproducibility of the LC-MS system we could exclude system inherent influence. The Pearson correlation of log_2_-expression value between the technical replicates on day 0 was 0.967. 

**Figure 3:**
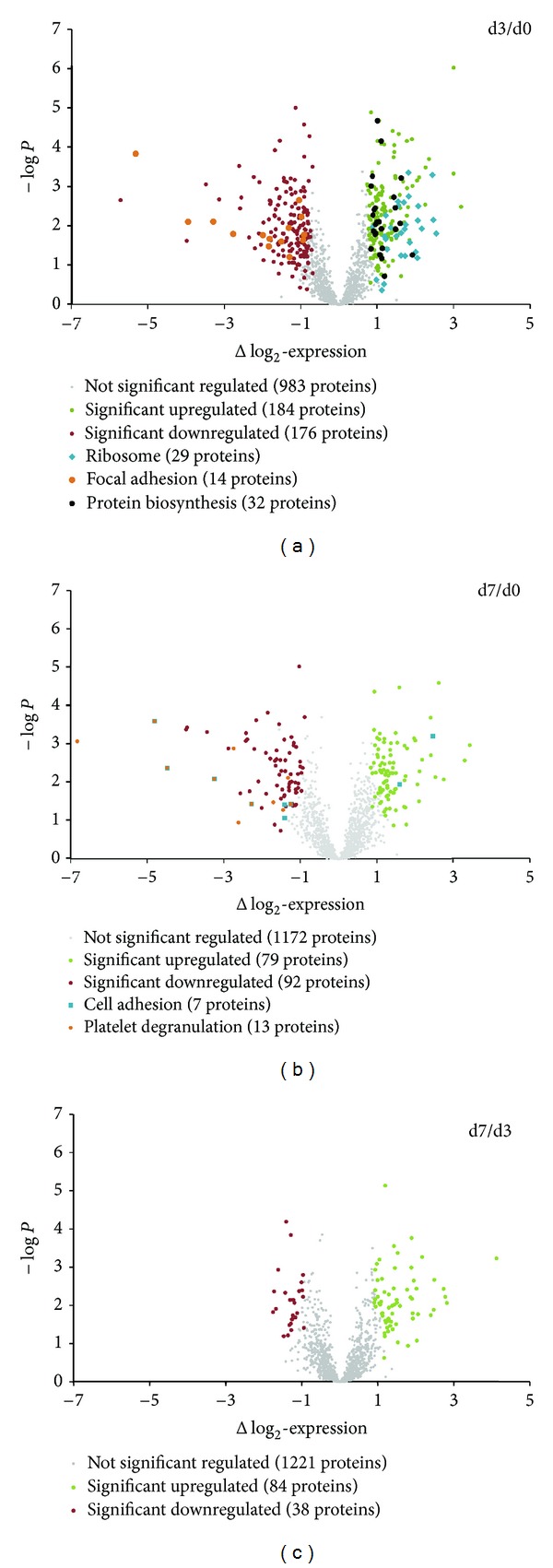
Volcano plot of pairwise differences in protein abundance. Significant regulated proteins must show differences in log_2_-differences in protein abundance (*x*-axis) and in the –log, *P* value (*y*-axis). Proteins with biological processes like focal adhesion or protein biosynthesis showed a strong regulation between day 0 and day 3.

Statistical analysis of protein abundance by ANOVA analysis of 1343 proteins revealed that 903 of the identified proteins (67.2%) showed no significant regulation at any time point, whereas 440 proteins showed significant changes along the analysed expansion time (Figure 3). In agreement with the PCA, we observed the greatest number of expression changes already after 3 days (time point d3) in an early stage of *ex vivo* expansion (Table 1). In total 360 proteins were significantly regulated after 3 days. From these proteins 318 proteins were also found significantly changed on day 7 and exhibited an “early-permanent” effect during *ex vivo* expansion. In comparison to this group of proteins we observed 42 proteins with “early-transient” abundance change. From day 3 to day 7 a small group of 80 proteins showed alterations in abundance level in late stage of *ex vivo* expansion. 

To describe the changes on protein level, we defined five different expression profiles (Table 2): “Early-transient” and “early-permanent” reflect early changes in the proteome of CD34^+^ haematopoetic stem/progenitor cells, while “late” as well as “long term” reflect changes during longer expansion time. Within the group of early transient upregulated proteins (in total 17 proteins) we found the transferrin receptor protein 1 (CD71) 7.6-fold upregulated on day 3 and 2.3-fold downregulated on day 7. An equal number of 36 proteins were detected to be long-term regulated (21 up, 15 down). Representative proteins are the actin-binding protein tropomodulin-3, the cyclin-dependent kinase 6 (both long term downregulated), or two tRNA-ligases responsible for amino acid activation (VARS and WARS, both long-term upregulated).

### 3.3. Biological Processes Involved in *Ex Vivo* Expansion

Next we were interested to extract the biological information of the proteome changes upon *ex vivo* expansion. Therefore, we searched for enriched Gene Ontology terms (molecular function and biological processes), KEGG-Pathways, and Uniprot Keywords within the regulated groups of proteins (Table 2, Figure 3).

Within the group of early permanent upregulated proteins biological processes linked to translation (initiation/elongation), ribosomal activity, and protein biosynthesis are significantly enriched. From 1343 proteins, 33 are linked with the KEGG-pathway “ribosome,” whereof 27 are up-regulated with a fold change of 1.85 to 5.51. Significant enrichment could also be found for the Uniprot Keyword “protein biosynthesis.” 31 proteins out of 64 proteins in the whole dataset showed a significant up-regulation. We identified several translation initiation factors (e.g., EIF2A, EIF3A, EIF4A, and EIF5B) as well as 10 tRNA-ligases in the aminoacyl-tRNA-biosynthesis pathway (e.g., GARS, MARS, and NARS) being permanently upregulated.

The proteins which were found permanently downregulated during *ex vivo* expansion revealed an enrichment of the KEGG-pathway focal adhesion (e.g., integrin *α*-2b/*β*-3 and filamin A/B), the GO-Term exocytosis as well as platelet degranulation (e.g., platelet basic protein). 

As the number of proteins regulated between days 3 and 7 was smaller compared to day 0/day 3 (Figure 3), less categories with proteins regulated only in a later stage during *ex vivo* expansion were significantly enriched. We observed cell adhesion molecules (HLA-DRA, ITGAL—known as CD11a, ITB2—known as CD18) as well as the general term of “glycoproteins” (e.g., CD74 and VIM) being enriched in the group of late time up-regulated proteins.

**Figure 4:**
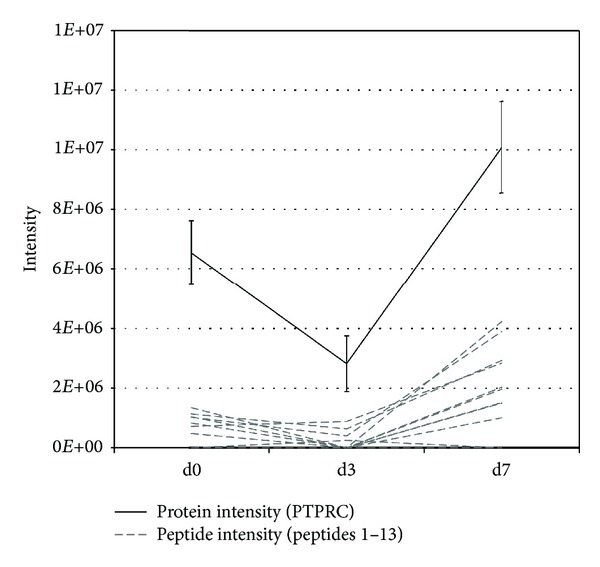
Expression of PTPRC. The labelfree intensity of PTPRC (also known as CD45) is quantified based on 13 unique peptides with similar expression profiles. A significant down regulation with a fold change of −2.33 was observed between day 0 and day 3. From day 3 to day 7 a significant up regulation with a fold change of +3.6 was observed.

Furthermore, we considered the group of protein transiently or long-term changed during *ex vivo* expansion. Due to a lower number of proteins in these groups, a significant enrichment of biological processes was not observed. Nevertheless, proteins with this expression profiles can be relevant for the understanding of *ex vivo* expansion of CD34^+^ cells. For example, we quantified receptor-type tyrosine-protein phosphatase C (PTPRC), also known as CD45. PTPRC was quantified based on 13 peptides with similar expression profiles (Figure 4) falling into the category of early transient down-regulated proteins. This protein showed a significant lower expression (fold change −2.3) on day 3, but interestingly the abundance of CD45 in the day 7 population was 3.6-fold higher than on day 3 and therewith PTPRC almost reached a higher abundance than at day 0. These findings are consistent with the observed number of CD45^+^ cells during FACS (Figure 1(a)), where the number of CD45^+^ cells was lower on day 3 compared to day 0 as well as day 7.

### 3.4. Cytometric Validation of Selected Surface Marker

For the validation of identified candidate proteins we performed flow cytometry of selected surface markers. The data for CD13 as well as for CD71 can be found in supplementary Figure 2. CD34^+^ cells displayed a medium expression of CD13 already on day 0 which significantly increased by day 3 and then slightly declined by day 7. CD71, on the other hand, was only weakly expressed on day 0, increased by day 3, and stayed stable up to day 7. These observations are congruent to the intensity values obtained by label-free quantification on proteome level, where CD13 shows an early upregulated profile and CD71 a long-term upregulated profile.

### 3.5. Influence of Cytokines Used for *Ex Vivo* Expansion

In our dataset we searched explicitly for proteins linked with the cytokines used for *ex vivo* expansion, in particular SCF, TPO, FLT3-lg, and IL-6. No permanent influence on direct interaction partners could be observed. We used STRING [21] and Ingenuity Pathway Analysis (Ingenuity systems, http://www.ingenuity.com/) to search for regulated downstream effectors or a general regulation of cytokine regulated pathways, but detected no significant changes on protein level, neither after 3 days nor after 7 days of expansion. The same is true for the NOTCH-signaling pathway, which is known to be linked with *ex vivo* expansion of CD45^+^ cells [5], where no regulation was observed. 

## 4. Discussion

Application of cytokine-supplemented media results in fast and effective expansion of haematopoietic cells [1]. In our experiments on the *ex vivo* expansion of cord-blood-derived CD34^+^ cells by application of cytokines, total cell count increased within the first 3 days by approximately factor 2. Nearly all cells still expressed the CD34-epitope on the surface, without any distinct morphological change or difference in flow cytometric analysis in comparison to native CD34^+^ cells. 

In-depth proteomics identified 2970 unique proteins in cell lysates stemming from *ex vivo* expanded CD34^+^ cells derived from umbilical cord blood. This is—to our knowledge—by now the most detailed view on protein level for these cells along *ex vivo* expansion time of seven days. Of 290 proteins described by [12] in native CD34^+^ cells we confirmed 221 proteins. Label-free quantification of 1343 proteins indicates major changes in the proteome along the expansion time. 

However, proteomic assessment revealed a significant change in the protein profile already at day three of expansion. In particular proteins that are related to the family of translation factors, ribosomal activity and cell cycle function are upregulated. This is in accordance with the increasing cell count observed. In contrast to this, early downregulated proteins are linked with specific focal adhesion/cytoskeleton remodelling functions (e.g., ACTN1, ITGB3, TLN1, and VASP). Although native CD34^+^ cells are of rather nonadherent character under *in vitro* culture conditions, they still bear the intrinsic potential to engraft into tissue (e.g., to home into bone marrow). Therefore, the decrease of proteins with extracellular matrix linkage might be explained by a loss of this property due to differentiation of the cells and subsequent reduced ability to engraft.

During continued expansion, the cell number increased further, but flow cytometric assessment on day 7 suggests a potential differentiation of the cells and showed that less than 20% of the cells still express CD34. Additionally the presence of a population of CD34^high^-expressing cells (Figure 1), which is specific for most mature leukocytes, was observed. This variation is also reflected by a change in the CD45-protein expression profile (Figure 4). It is interesting to note that in PCA, this shift does not result in a higher difference for day 3/day 7 compared to day 0/day 3 (as depicted in Figure 2). Although the expansion rate of CD34^+^ cells from day 0 to day 3 is on the same level than of day 3 to day 7 (Figure 1(b)), we conclude that the day-3 cells are already undergoing severe changes. Furthermore, most proteins and associated pathways were permanently changed over the analysed expansion time, as only minor changes between later time points were observed. As no regulation of direct interaction partners or downstream effectors of the cytokines used during expansion could be observed, we propose that the described changes could be more independent from the used expansion protocol than believed so far. Further insight might be gained by applying targeted proteomics techniques with a higher sensitivity against special pathways (e.g., NOTCH pathway).

Late effects on the protein level are in particular the higher abundance of specialised cell adhesion molecules. While the change in the CD pattern observed in flow cytometry (CD45-expression) on day 7 is confirmed by proteomics (not only of CD45 and CD34, but also CD11, CD18, and CD74), assessment of protein expression clearly demonstrates that severe changes occur much earlier.

Interestingly, several proteins within the late regulated group of proteins are linked with leukocyte or lymphocyte activation (DUSP3, HLA-DRA, PAK1, CD45, and SYK). Since a potential differentiation might result in an impaired ability to engraft in a patient, but might otherwise lead to a faster reconstitution, further examination of these effects is highly recommended. Future aspects should involve assessment of CD34 subpopulations, such as CD34^+^/CD38^−^ cells, which are considered as the most primitive haematopoetic progenitor, by resorting after expansion. It also should be examined, whether variations in cytokines applied can direct predifferentiation into specific directions (e.g., either myeloid or lymphoid).

## 5. Conclusion

*Ex vivo* expansion of cord-blood-derived CD34^+^ cells by application of cytokine-supplemented media results in fast and effective expansion of haematopoetic cells and therefore is an interesting approach that is intensively pursued. Proteomic profiling of expanded cells revealed early upregulation of proteins related to protein biosynthesis but downregulation of focal adhesion, exocytosis proteins within three days.

Though, it can be argued that this procedure may alter the properties of the haematopoetic stem/progenitor cells. While a direction on day 3 could not be distinguished based on our data, there are indications for a general leukocyte and/or lymphocyte differentiation in further cultivated cells, which is not accessible by flow cytometry.

Taking this into account, questions arise concerning the effectiveness of the expansion in regard to maintenance of the undifferentiated state of the haematopoetic cells.

## Supplementary Material

Supplemental table 1: List of quantified proteins with expression profiles from day 0 to day 7.
Supplemental table 2: List of all identified proteins, peptides, peptides with modification, label free quantification values.
Supplemental figure 1: Expansion of total cells, CD34+-cells and CFC in a preliminary experiment.
Supplemental figure 2: Validation of protein quantification values via flow cytometry (FCM).

## Figures and Tables

**Table 1:** Number of proteins with expression profiles from day 0 to day 7. Discrimination between early, late, and long-term changes as well as between transient and permanent regulations shows that most significant changes occur early and remain regulated permanently.

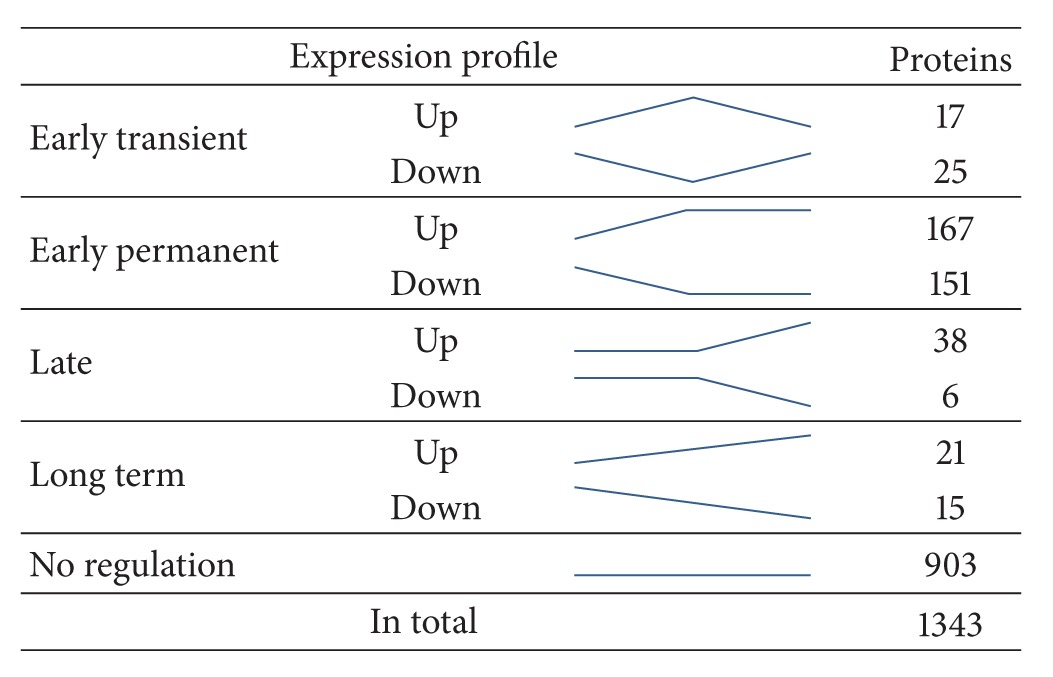

**Table 2:** Examples of enriched biological processes in significant regulated proteins.

Category	Number of proteins regulated (in total)	Enrichment factor	*P* value	Regulated proteins
	Early permanent: upregulated			

Serine family amino acid metabolic process^1^	4 (4)	8.04	2.3*E* − 04	PHGDH; PSPH; SHMT1; SHMT2
Translational initiation^1^	28 (34)	6.62	3.7*E* − 21	EIF2B2; EIF2B4; EIF3B; EIF3E; EIF3H; EIF3K; EIF4G1; EIF4G2; EIF5B; PABPC1; RPL12; RPL13; RPL14; RPL17; RPL18; RPL22; RPL27; RPL3; RPL38; RPL4; RPL7; RPL7A; RPL9; RPLP0; RPLP0P6; RPS10; RPS10P5; RPS13; RPS16; RPS18; RPS19; RPS2; RPS25; RPS3; RPS3A; RPS4X; RPS7; RPS8; RPSA; RPSAP58
Ribosome^2^	27 (33)	6.58	2.9*E* − 20	PWP2; RPL12; RPL13; RPL14; RPL17; RPL18; RPL22; RPL27; RPL3; RPL38; RPL4; RPL7; RPL7A; RPL9; RPLP0; RPLP0P6; RPS10; RPS10P5; RPS13; RPS16; RPS18; RPS19; RPS2; RPS25; RPS3; RPS3A; RPS4X; RPS7; RPS8; RPSA; RPSAP58
Translational elongation^1^	28 (35)	6.43	1.7*E* − 20	GFM1; RPL12; RPL13; RPL14; RPL17; RPL18; RPL22; RPL27; RPL3; RPL38; RPL4; RPL7; RPL7A; RPL9; RPLP0; RPLP0P6; RPS10; RPS10P5; RPS13; RPS16; RPS18; RPS19; RPS2; RPS25; RPS3; RPS3A; RPS4X; RPS7; RPS8; RPSA; RPSAP58
Cellular process involved in reproduction^1^	29 (42)	5.55	3.4*E* − 18	CDK1; RPL12; RPL13; RPL14; RPL17; RPL18; RPL22; RPL27; RPL3; RPL38; RPL4; RPL7; RPL7A; RPL9; RPLP0; RPLP0P6; RPS10; RPS10P5; RPS13; RPS16; RPS18; RPS19; RPS2; RPS25; RPS3; RPS3A; RPS4X; RPS7; RPS8; RPSA; RPSAP58; UBAP2L
Protein targeting to ER^1^	27 (40)	5.43	1.4*E* − 16	RPL12; RPL13; RPL14; RPL17; RPL18; RPL22; RPL27; RPL3; RPL38; RPL4; RPL7; RPL7A; RPL9; RPLP0; RPLP0P6; RPS10; RPS10P5; RPS13; RPS16; RPS18; RPS19; RPS2; RPS25; RPS3; RPS3A; RPS4X; RPS7; RPS8; RPSA; RPSAP58
Initiation factor^3^	17 (29)	4.71	2.4*E* − 09	EIF2A; EIF2B2; EIF2B4; EIF2S1; EIF2S2; EIF3A; EIF3B; EIF3C; EIF3CL; EIF3D; EIF3E; EIF3EIP; EIF3L; EIF3H; EIF3K; EIF4A1; EIF4G1; EIF4G2; EIF5B
Protein biosynthesis^3^	31 (64)	3.90	2.9*E* − 13	AARS; CARS; EEF1A1; EEF1A1P5; EEF1A2; EEF1D; EEF1G; EEF2; EIF2A; EIF2B2; EIF2B4; EIF2S1; EIF2S2; EIF3A; EIF3B; EIF3C;EIF3CL; EIF3D; EIF3E; EIF3EIP; EIF3L; EIF3H; EIF3K; EIF4A1; EIF4G1; EIF4G2; EIF5B; EPRS; ETF1; GARS; GFM1; IARS; MARS; NARS; TARS
Ribonucleoprotein^3^	28 (78)	2.89	2.2*E* − 08	MRPS27; RPL12; RPL13; RPL14; RPL17; RPL18; RPL22; RPL27; RPL3; RPL38; RPL4; RPL7; RPL7A; RPL9; RPLP0; RPLP0P6; RPS10; RPS10P5; RPS13; RPS16; RPS18; RPS19; RPS2; RPS25; RPS3; RPS3A; RPS4X; RPS7; RPS8; RPSA; RPSAP58
RNA transport^2^	22 (66)	2.68	3.4*E* − 06	EEF1A1; EEF1A1P5; EEF1A2; EIF2B2; EIF2B4; EIF2S1; EIF2S2; EIF3A; EIF3B; EIF3C; EIF3CL; EIF3D; EIF3E; EIF3H; EIF4A1; EIF4G1; EIF4G2; EIF5B; ELAC2; GEMIN5; NUP93; PABPC1; PABPC4; RANGAP1; XPOT
Cellular macromolecule catabolic process^1^	43 (145)	2.38	2.0*E* − 09	CDK1; EIF3E; EIF4A1; EIF4G1; ETF1; FEN1; PABPC1; PABPC4; PSMC1; PSMC2; PSMD1; PSMD2; PSMD3
ncRNA metabolic process^1^	21 (71)	2.38	4.3*E* − 05	AARS; CARS; ELAC2; EPRS; FTSJ3; GARS; GEMIN5; IARS; MARS; NARS; NOP2; PDCD11; RPL14

	Early permanent: downregulated			

Platelet degranulation^1^	13 (21)	5.43	2.8*E* − 08	ACTN1; CALM2; F13A1; FLNA; ITGA2B; ITGB3; PECAM1; PPBP; THBS1; TLN1; TUBA4A; VCL; WDR1
Focal adhesion^2^	13 (22)	5.19	6.2*E* − 08	ACTN1; CRKL; FLNA; FLNB; ITGA2B; ITGB3; MYL12A; PPP1R12A; RAP1B; THBS1; TLN1; VASP; VCL
Exocytosis^1^	14 (24)	5.12	2.3*E* − 08	ACTN1; CALM2;CALM1; F13A1; FLNA; ITGA2B; ITGB3; PECAM1; PLEK; PPBP; SCRN1; SNCA; THBS1; TLN1; TUBA4A; VCL; WDR1
Calmodulin binding^3^	6 (11)	4.79	5.2*E* − 04	ADD3; IQGAP2; PPP3CA; SPTAN1; SPTBN1; STRN
Organic acid catabolic process^1^	14 (38)	3.23	2.4*E* − 05	ACADM; ALDH6A1; BCKDHA; CPT2; DDAH2; DECR1; DLST; ECI1; DCI; ETHE1; GLUD1; GLUD2; HADHB; HIBCH; IVD; MCCC1
Cell activation^1^	16 (48)	2.93	2.6*E* − 05	ACTN1; ADA; CALM2; CALM1; F13A1; FLNA; ITGA2B; PECAM1; PLEK; PPBP; PPP3CA; RAP1B; RAP1A; THBS1; TLN1; TUBA4A; VCL; WDR1; YWHAZ

	Late regulated: upregulated			

Cell adhesion molecules (CAMs)^2^	3 (5)	21.21	2.0*E* − 04	HLA-DRA; ITGAL; ITGB2;
Glycoprotein^3^	11 (76)	5.12	2.9*E* − 06	ASAH1; CD74; GLB1; HLA-DRA; ITGAL; ITGB2; MPO; PLD3; PRTN3; RNASET2; VIM;
Signal^3^	9 (67)	4.75	5.0*E* − 05	ASAH1; IL-25; CD74; GLB1; HLA-DRA; ITGAL; ITGB2; MPO; PLD3; PRTN3; RNASET2

	Late regulated: downregulated			

No significant enriched GO-Terms due to protein number <30				

	Long term: up- and downregulated			

No significant enriched GO-Terms due to protein number <30				

	Early transient: up- and downregulated			

No significant enriched GO-Terms due to protein number <30				

Annotations for biological processes (GOBP^1^) KEGG^2^, and Uniprot Keywords^3^ of regulated proteins were compared with proteins in the whole dataset. Enriched categories have at least an enrichment factor >2.3 and a *P* value of less than 0.001. Due to multiple functionalities of proteins, some proteins are listed in multiple categories.
