# Supplementary material for: Proteomic Profiling of Ex Vivo Expanded CD34-Positive Haematopoetic Cells Derived from Umbilical Cord Blood
Source: Stem Cells Int. 2013 Mar 26;2013:245695. doi: 10.1155/2013/245695 (PMC3622389; doi:10.1155/2013/245695)

Label free quantification

(normalised intensity)

10

2

10

3

10

4

10

5

CD13 PE

0

10

20

30

40

50

60

70

80

Count

d7


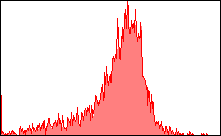


**CD13**

**(ANPEP)**

10

2

10

3

10

4

10

5

CD13 PE

0

100

200

300

400

500

600

Count

d0


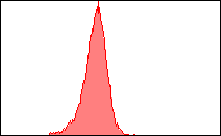


10

2

10

3

10

4

10

5

CD13 PE

0

50

100

150

200

250

300

Count

d3


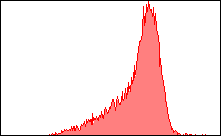


10

2

10

3

10

4

10

5

CD71 FITC

0

50

100

150

200

250

Count

d3


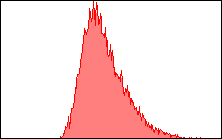


10

2

10

3

10

4

10

5

CD71 FITC

0

10

20

30

40

50

60

70

Count

d7


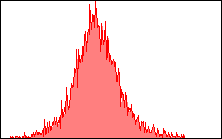


P5

**CD71**

**(TFRC)**

10

2

10

3

10

4

10

5

CD71 FITC

0

50

100

150

200

250

300

350

Count

d0


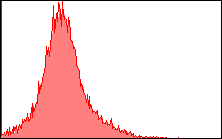

Supplement: Supplementary file 3 [file 245695.f3.docx]
