# Supplementary material for: Proteomic Profiling of Ex Vivo Expanded CD34-Positive Haematopoetic Cells Derived from Umbilical Cord Blood
Source: Stem Cells Int. 2013 Mar 26;2013:245695. doi: 10.1155/2013/245695 (PMC3622389; doi:10.1155/2013/245695)
Supplement: Supplementary file 3 [file 245695.f2.docx]

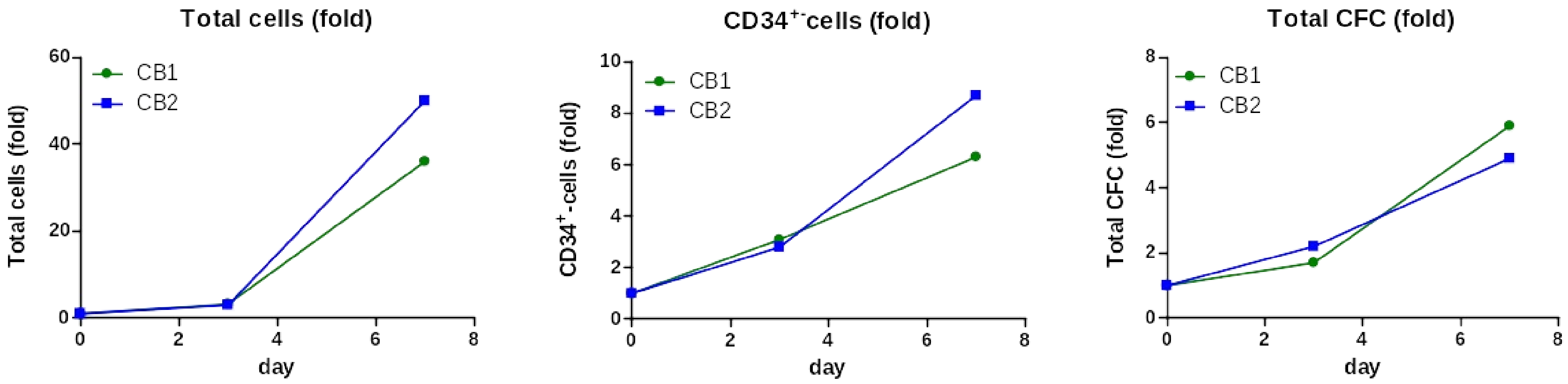

Total cells (fold)
CB1
CB2
Total cells (fold)
0
20
40
60
0
2
4
6
8
day
CD34^{+}-cells (fold)
CB1
CB2
CD34^{+}-cells (fold)
0
2
4
6
8
10
0
2
4
6
8
day
Total CFC (fold)
CB1
CB2
Total CFC (fold)
0
2
4
6
8
0
2
4
6
8
day
